# The Bradshaw Lecture

**Published:** 1902-11-15

**Authors:** 


### THE BRADSHAW LECTURE.

As the theme of the Bradshaw lecture, delivered before the Royal College of Physicians of London, Dr. Charles J. Cullingworth chose the subject of "Intraperitoneal Hæmorrhage Incident to Ectopic Gestation." Part of the lecture was historical, part —and a most interesting part—was devoted to a lucid account of the symptoms met with in these cases of diffuse intraperitoneal hæmorrhage due to ectopic gestation and on the diagnosis of the condition. Then the lecturer came to the treatment to be adopted, showing how even in the most desperate condition women had been snatched from death by removing the ruptured and bleeding tube.

Then he turned to the non-diffuse or encysted forms of intraperitoneal hæmorrhage—in other words pelvic hæmatocele—describing the symptoms and meaning of this condition, and the results of its rupture when this takes place. Finally he described the modes of origin of these two forms of hæmorrhage, the diffuse and the non-diffuse, and here we cannot do better than quote his own words, with such omissions as exigencies of space demand. The particular disturbance to which for some time both these varieties of intra-peritoneal hæmorrhage, when connected at all with ectopic gestation, were held to be invariably due was rupture of the gestation sac. The rupture was supposed to be brought about by the Fallopian tube becoming stretched and thinned by the growing ovum until the thinning of the tube at that part became so extreme that a comparatively slight strain sufficed to cause it to give way. But it gradually became evident that the simple explanation of the liability of an ectopic gestation to undergo rupture did not take into account all the factors that were usually concerned in the production of rupture. Observers began to call attention to the frequency with which rupture was found to have been preceded by the occurrence of hæmorrhage within and around the ovum itself. Later still it was realised that intra-peritoneal hæmorrhage in connection with ectopic gestation might occur without rupture, the blood escaping not through a rent, but from the free end of the pregnant tube. And eventually it was found that whereas diffuse intra-peritoneal hæmorrhage was in the majority of cases due to tubal rupture and only occasionally to leakage from the open fimbriated end of the tube, the encysted form of intra-peritoneal hæmorrhage, or pelvic hæmatocele, was most frequently due to leakage from the mouth of the tube, and only in a comparatively small number of cases to rupture. In other words, it was observed that the ordinary result of tubal rupture is a diffuse and highly dangerous intra-peritoneal hæmorrhage, whilst the ordinary result of bleeding from the mouth of the tube is the formation of a hæmatocele.

In the vast majority of cases of ectopic gestation the gestation begins in the Fallopian tube. Up to a very recent date the statement that every ectopic gestation is, or began by being, tubal would have been accepted as correct. But within the last few years several indisputable instances of ovarian pregnancy have been recorded, so that such a statement can no longer be made. Nevertheless, it remains true that, with very few exceptions, an ectopic gestation means a tubal gestation.

The fertilised ovum may be arrested in any part of the Fallopian tube. The subsequent fate of the fertilised ovum is largely determined by the site of the gestation. When it is arrested, for instance, within the uterine wall its comparatively early death appears to be all but inevitable, and when it is arrested immediately outside the uterus its life appears to be almost equally, if not more precarious. In the middle and outer portions it has a better chance, but even here it succumbs in the great majority of cases to one or other of the dangers that threaten it and it is rare for its development to continue long enough for the fœtus to become viable and still rarer for it to continue up to full term. In whatever part of the tube the fertilised ovum lodges a localised swelling is produced, slight at first, but gradually increasing in size with the size of the ovum. Simultaneously there occurs a general hyperæmia of the pregnant tube and of the parts in immediate connection with it, all the vessels of the neighbourhood undergoing an increase in size. The tube wall seldom becomes hypertrophied. The usual result, indeed, is for it to become attenuated as it is expanded by the growing ovum which, of course, it completely and closely surrounds. But the chief interest centres in the mode of attachment of the ovum. Though everywhere in contact with the tube wall, it only becomes actually attached to it over a limited area. This area forms the site of the future placenta, the chorionic villi which at first completely surround the ovum becoming specially developed there and penetrating the tube wall even up to its outer coat. The result of this ingrowing of the chorionic villi is that the muscular fibres become separated and the tube wall becomes at that spot sensibly weakened. The separation of the muscular fibres is still further increased by an infiltration of large nucleated cells. Meanwhile a membrane, which all authorities agree in regarding as a membrana decidua, is being formed in the uterine cavity and the uterus itself is undergoing enlargement. This enlargement continues if the ovum lives for the first two or three months much as it would do if the gestation were intra-uterine. But about the end of that time the uterus appears to find out that it has been cheated and ceases to grow, though as long as there is a living and growing ovum in its neighbourhood it does not retrieve its blunder—does not, in in other words, commence to undergo the process of involution.

It will be easily understood that rupture of the gestation sac is specially apt to occur at the spot where the tube wall is weakened and where it is often not only weakened but much thinned. Cases have been observed where nothing intervened between the ovum and the peritoneal covering of the tube wall but a little newly-organised connective tissue together with some of the cellular elements already mentioned. One case is described where a partially-organised blod-clot lying in the intervillous space and covered by peritoneum was all that remained to represent the tube wall at the seat of rupture. It is evident from what has been said that processes are at work which greatly predispose to rupture. A little extra strain upon the capacity of the intervillous spaces containing the maternal blood, or the occurrence, by no means infrequent, of an interstitial hæmorrhage, is quite enough to bring about a catastrophe. In some cases the catastrophe is for a time averted by the formation of an inflammatory deposit on the outer surface of the peritoneum over the placental area, but this is seldom more than a temporary safeguard. Such a layer of adventitious tissue is not sufficiently elastic to bear any considerable amount of stretching and before long it, too, gives way and the rupture is complete. Probably, owing to the lumen of the tube being at that part excessively fine and its walls dense and inelastic so that its limits of expansion are soon reached, rupture is apt to take place with special frequency and at a particularly early stage of gestation in cases where the ovum is arrested in the straight portion of the tube just external to the uterus.

Another factor in the etiology of intra-peritoneal hæmorrhage, a factor which has not until the last few years been fully appreciated, is to be found in certain accidental changes which are apt to occur in the ovum itself. The ovum in tubal gestation is specially prone to become the seat of hæmorrhage and to have its bulk enlarged thereby and its vitality destroyed. Sometimes the result is the formation of something so like the well-known carneous mole of uterine pregnancy that it may be appropriately termed a tubal mole. At other times the amniotic cavity is so much compressed by the hæmorrhagic effusion between the membranes that it is flattened and practically obliterated, the embryo being destroyed, and the ovum, whilst retaining its shape and often retaining its attachment, becoming a mere mass of clot and *débris*. This is, in my experience, a much more frequent result than the formation of a typical mole with its distorted or blighted embryo, its distinct amniotic cavity, and its little rounded bosses of blood clot projecting beneath its lining membrane. Not infrequently the covering of the ovum gives way and the tube becomes filled with clot and *débris*, all trace of ovum being lost. This constitutes one of the varieties of hæmatosalpinx. The sudden increase in the bulk of the ovum due to this hæmorrhage is very often a factor in the bringing about of rupture. But hæmorrhage into the ovum—apoplexy of the ovum, as it is called—does non always result in the production of rupture. It does not even do so in the majority of cases. The more usual result is a trickling of blood from the abdominal end of the Fallopian tube. Now such a trickling exactly fulfils the conditions necessary to the formation of a hæmatocele. The blood flows so slowly and in such moderate amount that it has time to collect in the pelvis and to become encysted. Of course, there are exceptional cases, but the ordinary result of bleeding from the open fimbriated end of a pregnant Fallopian tube is the formation of a hæmatocele. The capsule of a typical pelvic hæmatocele is formed partly by the peritoneal surfaces of the various surrounding parts (the broad ligament, the uterus, the ovary, the appendages of the opposite side, the intestines, the omentum, and the walls and floor of the pelvis) and partly by an adventitious cyst wall which consists of a delicate connective-tissue basis upon which successive layers of fibrin have been deposited from within. The layers of fibrin gradually deposited upon it are derived from the contents of the hæmatocele. The wall thus constituted tends to become firmer and harder day by day, and it was this solidifying and gradual hardening of the periphery that led to the old supposition that the hæmatocele, soft and elastic, if not, indeed, actually fluid at first, coagulates *en masse* and gradually hardens until it becomes for all practical purposes a solid tumour. As a matter of fact, the inner and main portion of the hæmatocele almost invariably remains either in a fluid condition or in the condition of soft clot.

The abdominal end of the tube is differently affected according to whether the ovum has lodged in the inner or outer part of the tube. When the isthmus of the tube—*i.e.*, the inner straight portion —is the seat of the gestation the fimbriated mouth of the tube is sufficiently far from the scene of activity to remain, as a rule, unaffected. But in the much more common event of the gestation occurring in the outer portion of the tube the abdominal ostium remains open until from the sixth to the eighth week and sometimes becomes abnormally expanded. After that time it usually becomes closed, or nearly closed, by adhesions, provided the gestation has continued to that date without interruption. It is during those earlier weeks, when the abdominal ostium is still patulous, that a hæmatocele is most apt to be formed. For it is during the first two months of gestation that hæmorrhage within and around the ovum is most liable to occur, and the abdominal ostium remaining patent the blood extravasated around the ovum usually finds vent in that direction. But owing to the changes that I have described in the tube wall rupture may take place, and does not infrequently take place, while the abdominal ostium is still open, and not a few cases have been recorded in which bleeding has occurred both from rupture of the tube wall and from the mouth of the tube. It will also be easily understood that under the pressure of recurrent hæmorrhages or from any undue strain the sac of a hæmatocele may give way and bleeding may take place into the general peritoneal cavity. In other words, a case of encysted intra-peritoneal hæmorrhage may suddenly become converted into the more dangerous diffuse form. This should be borne in mind during the bimanual examination of such cases for diagnostic purposes, especially when such an examination is conducted under anæsthesia. I have seen several cases in which this accident has happened and in which there was no reasonable doubt as to its having been caused by diagnostic manipulation.

Time will not permit of my following at any length the fortunes either of a pelvic hæmatocle or of a diffuse but non-fatal intra-peritoneal hæmorrhage. The tendency in both cases is to gradual absorption of the extravasated blood and to the recovery of the patient. But there are pitfalls in the way, and of these the two most important are the liability to fresh hæmorrhages and the liability to bacterial infection of the blood already poured out. Whenever an apoplectic ovum is retained and adherent within the Fallopian tube a woman is exposed to the constant risk of recurrent hæmorrhage.

